# Multidimensional Diversity Identifies Mountains as Key Refugia for Caudata in China

**DOI:** 10.1002/ece3.73497

**Published:** 2026-05-13

**Authors:** Lu‐Lu Sui, Bin Wang, Feng Xie, Wei Zhu, Cheng Shen, Jian‐Ping Jiang

**Affiliations:** ^1^ Mountain Ecological Restoration and Biodiversity Conservation Key Laboratory of Sichuan Province, Chengdu Institute of Biology Chinese Academy of Sciences Chengdu China; ^2^ China‐Croatia Belt and Road Joint Laboratory on Biodiversity and Ecosystem Services, Chengdu Institute of Biology Chinese Academy of Sciences Chengdu China; ^3^ University of Chinese Academy of Sciences Beijing China; ^4^ Mangkang Biodiversity and Ecological Station Tibet Ecological Safety Monitor Network Changdu China

**Keywords:** Caudata, climatic refugia, functional diversity, phylogenetic diversity, taxonomic diversity

## Abstract

Climate change is reshaping biodiversity patterns, yet conservation planning for amphibians, particularly Caudata, remains limited in both geographic coverage and the integration of multiple biodiversity dimensions. In this study, we combined species distribution models (SDMs), taxonomic, phylogenetic, and functional dimensions of biodiversity, and Zonation‐based spatial prioritization to identify conservation priorities for Caudata in China. The analysis includes species with limited occurrence data, allowing a more complete representation of the group. Multidimensional diversity shows a clear spatial structure, forming three distinct biogeographic clusters, with hotspots mainly concentrated in mountainous and karst regions. Phylogenetic and functional diversity display strong spatial congruence, suggesting that functional differentiation is largely conserved across evolutionary lineages. In contrast, overlap among priority areas based on different biodiversity dimensions is limited, and only 20.23% of grid cells are identified as high priority across all three dimensions. Current protected areas cover only a small proportion of high‐diversity regions, ranging from 7.35% to 12.57%, and this coverage is projected to decline under future climate scenarios. Although priority areas shift to some extent, their spatial patterns remain largely consistent across scenarios, with an overlap of 76.79%–84.99%. Based on this stability, we identify 14 climate‐stable refugia that represent key areas for long‐term conservation, yet only about 9% of these areas are currently protected. These results indicate that mountain systems play a dual role as centers of present‐day diversity and as refugia under future climate change. Identifying spatially stable priority areas provides a practical basis for improving protected area networks, strengthening connectivity along elevational gradients, and integrating climate change into conservation planning. This framework may also support the prioritization of other climate‐vulnerable and data‐poor taxa.

## Graphical abstract

We identified 14 critical refugia as conservation gaps, emphasizing mountain ecosystems' buffering capacity for Caudate species. Our findings highlight the urgency of adaptive conservation strategies targeting climate refugia and expanding PA networks.

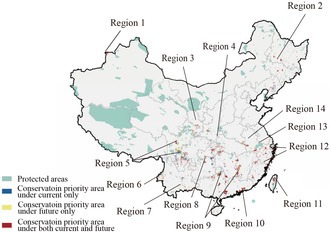

## 1 Introduction

Climate change is reshaping global biodiversity patterns and remains a central challenge in conservation biology (Wake and Vredenburg 2008; Murali et al. 2023; Urban 2024). Mounting evidence shows that warming‐driven range shifts, habitat fragmentation, and phenological mismatches are not uniform across taxa but disproportionately affect ecologically sensitive lineages (Pecl et al. 2017; Yin et al. 2023). In this context, protected areas (PAs) are often considered potential climate refugia by maintaining ecosystem integrity and facilitating species dispersal through ecological corridor networks (Cazalis et al. 2020; Mi, Ma, et al. 2023). The Kunming–Montreal “30 × 30” target further underscores the urgency of expanding and improving PA networks (CBD 2022). Yet conservation planning and syntheses have largely prioritized highly speciose and charismatic vertebrate groups (Hu et al. 2021; Mi, Ma, et al. 2023; Mi, Song, et al. 2023; Zhang et al. 2023), leaving less‐studied but ecologically distinctive clades such as Caudata comparatively underrepresented, despite their strong microhabitat dependence, limited dispersal, and high sensitivity to climate and land‐use change.

Caudata are highly dependent on fine‐scale thermal and moisture regimes due to their permeable skin, non‐amniotic eggs, and biphasic life cycles that span headwater streams and forest‐floor microhabitats (Wells 2007). These traits make them acutely sensitive to microclimatic fluctuations and constrain their capacity to respond to environmental change. Most species exhibit low vagility and strong site fidelity, with effective dispersal rarely exceeding a few kilometers (Smith and Green 2005). In contrast, the mean velocity of climate change projected for the 21st century is approximately 0.42 km/year globally, frequently surpassing amphibians' potential tracking rates (Loarie et al. 2009). Beyond their vulnerability, Caudata play pivotal ecological roles in mediating energy flow and nutrient cycling across aquatic–terrestrial boundaries (Hocking and Babbitt 2014). Their dual life histories make them sensitive indicators of both freshwater and forest ecosystem integrity (Luedtke et al. 2023). Despite their functional importance and evolutionary distinctiveness, Caudata have received comparatively little conservation attention relative to other vertebrate groups (Mi, Ma, et al. 2023; Zhang et al. 2023). China represents a global center of Caudata diversity and phylogenetic endemism (Yao et al. 2018; Table S1), yet the conservation urgency of this lineage remains disproportionate: 65.3% of Chinese species are listed as State Key Protected Wild Animals (2021), 46.5% under CITES (2023), and 57.4% as IUCN‐threatened (IUCN 2025). Although research interest is increasing, the capacity of existing conservation frameworks to accommodate climate‐induced range shifts in these species remains poorly understood.

Effective biodiversity conservation requires preserving not only species but also their evolutionary heritage and ecological functions (Rodrigues 2006). The goal relies on a systematic understanding of the multidimensional nature of biodiversity. Traditional conservation planning has predominantly focused on taxonomic diversity (TD), using indicators such as species richness and endemism to identify priority areas (Brum et al. 2017). However, relying solely on TD is insufficient to capture the unique evolutionary trajectories or the functional heterogeneity of ecosystems (Mazel et al. 2018). To address this limitation, the incorporation of phylogenetic diversity (PD) and functional diversity (FD) has become an important advancement in modern conservation frameworks (Faith 1992; Bongers et al. 2021). FD describes differences among species in traits related to morphology, physiology, and ecological strategies, and thus reflects how species contribute to ecosystem functioning (Petchey and Gaston 2002). PD, in contrast, represents the evolutionary relationships among species and represents the accumulation of evolutionary history across lineages (Faith 1992; Webb et al. 2002; Schweiger et al. 2008). Compared with TD, these two dimensions provide complementary information, as they are more closely linked to ecosystem processes and stability (Cadotte et al. 2009, 2012; Campos et al. 2017). A multidimensional framework that jointly considers TD, PD, and FD is increasingly recognized as essential for comprehensive biodiversity assessment and effective spatial prioritization (Brum et al. 2017; Xu et al. 2019; Wang et al. 2023). This perspective is particularly critical under accelerating climate change, where different biodiversity dimensions often display spatial incongruence and distinct sensitivities to environmental shifts. Integrating these dimensions can improve the identification of climatic refugia and support more adaptive conservation strategies. This is particularly important for Caudata, whose evolutionary histories, ecological traits, and strong dependence on local environmental conditions make them especially sensitive to climatic change. Nevertheless, such multidimensional analyses remain scarce for Caudata, despite their ecological and evolutionary significance.

In this study, we integrated species distribution models (SDMs) with phylogenetic and functional analyses to map multidimensional diversity patterns and guide spatial conservation prioritization for Caudata in China under current and future climates. Specifically, we quantified the spatial patterns of taxonomic, phylogenetic, and functional diversity, identified priority conservation areas across these dimensions, and evaluated their overlap with existing protected areas and their potential as climatic refugia. By integrating multiple dimensions of biodiversity, this study provides a more comprehensive basis for conservation planning than species‐richness approaches alone. In particular, we identify spatially stable refugia across climate scenarios, highlighting areas that may serve as robust priorities for long‐term conservation of Caudata in China.

## 2 Methods

### 2.1 Species Composition

Based on three databases and book—Amphibia China (AmphibiaChina 2025), Amphibian Species of the World (Frost 2024) and China's Red List of Biodiversity (Jiang et al. 2021)—we compiled a preliminary list of Caudata in China. The list includes 101 species belonging to three families: Cryptobranchidae (1 genus, 4 species), Hynobiidae (9 genera, 33 species), and Salamandridae (8 genera, 64 species). This represents only 14.3% of the country's amphibian species diversity (Table S1, data as of March 31, 2025).

### 2.2 Species Occurrence Data Compilation and Cleaning

The occurrence data of Caudata in China was compiled from four sources: (i) our field survey data, (ii) published books and scientific literature (Table S2), (iii) specimen information from Chengdu Institute of Biology, Chinese Academy of Sciences, (iv) online database (GBIF, https://www.gbif.org). To ensure the highest data quality and taxonomic accuracy, we implemented a rigorous multi‐step screening and correction process. First, all species names were meticulously cross‐checked against the most recent authoritative classifications (Jiang et al. 2021; Frost 2024; AmphibiaChina 2025). Records associated with outdated synonyms or undergoing recent taxonomic revisions were corrected to reflect current accepted nomenclature. Then, the R package *CoordinateCleaner* v3.0.1 (Zizka et al. 2019) was used to filter out coordinates, removing records identified as outliers, country/province centroids, open ocean points, the GBIF headquarters, urban areas, or locations of biodiversity institutions (museums, zoos, botanical gardens, universities). Next, to mitigate spatial autocorrelation and prevent information redundancy, only one occurrence record per species was retained within each grid cell using ENMTools v1.3 (Warren et al. 2010). Last, 1706 occurrence records for all Caudata species in China were retained for further analyses.

### 2.3 Environmental Variables

We selected five types of environmental variables that could potentially affect Caudata distribution as candidate variables, including bioclimate, ecosystem function, topography, human impact, and habitat conditions. The current bioclimate and ecosystem function data were obtained from CHELSA v2.1 database (https://www.chelsa‐climate.org) for the period 1981–2010. A total of 19 bioclimatic variables were obtained, which primarily represented the annual trends or extreme factors of temperature and precipitation. Ecosystem function data were characterized using growing season length (GSL) and net primary productivity (NPP), providing insights into ecosystem dynamics and productivity. Topographic variables, including altitude, slope, and aspect, were derived from the digital elevation model (DEM) provided by WorldClim v2.1 (https://www.worldclim.org). Human impact was characterized using the latest human footprint and human population density (Center for International Earth Science Information Network 2018) from NASA Socioeconomic Data and Applications Center (SEDAC) (http://sedac.ciesin.columbia.edu/data/sets/browse). Habitat conditions were characterized using plant functional types (PFT) (Chen et al. 2022), as well as the mean UV‐B of highest month and mean UV‐B of lowest month (Beckmann et al. 2014), reflecting land types, seasonal UV‐B extremes, and baseline exposure levels. All variables were standardized to a 30‐arc‐second spatial resolution (~1 km × 1 km at the equator). Variables with significant contributions to models and low multicollinearity (Pearson correlation coefficients, |*r*| < 0.8) were retained (Figure S1), resulting in 16 variables for further analysis: mean diurnal temperature range (Bio2), mean temperature of wettest quarter (Bio8), mean temperature of coldest quarter (Bio11), precipitation of wettest month (Bio13), precipitation seasonality (Bio15), precipitation of driest quarter (Bio17), precipitation of warmest quarter (Bio18), precipitation of coldest quarter (Bio19), altitude, slope, aspect, GSL, human footprint, human population density, PFT and the mean UV‐B of the highest month.

Climate predictions for the 2050s (2041–2070 periods) and the 2100s (2071–2100 periods) across three Shared Socioeconomic Pathways (SSPs; SSP126, SSP370, and SSP585) were used to project future species distribution. By averaging the climate projections from these three Global Circulation Models (GFDL‐ESM4, MPI‐ESM1‐2‐HR, and MRI‐ESM2) for each grid cell, we aimed to reduce inter‐model variability (Jiang et al. 2023; Mi, Ma, et al. 2023). Future PFT layers corresponding to 2050 and 2100 were extracted from the same SSP framework.

### 2.4 Species Distribution Modeling

Three modeling strategies were employed according to the number of available species occurrences. For 49 species with at least 6 occurrence records, species distribution models (SDMs) were projected for each species using the R package *sdm* v1.1–8 (Naimi and Araújo 2016). To reduce algorithmic bias and improve model robustness, an ensemble modeling approach was employed, incorporating seven widely used SDM algorithms: the generalized linear model (GLM), generalized boosting model (GBM), maximum entropy model (MAXENT), random forest model (RF), support vector machine model (SVM), multivariate adaptive regression splines model (MARS), and flexible discriminant analysis model (FDA). For each species, the models were trained with randomly selected 70% of the distribution data and tested with the remaining 30%. To mitigate potential biases arising from the data, we implemented 5‐fold cross‐validations and repeated this process five times. Model performance was evaluated using the True Skill Statistic (TSS) and Area Under the Receiver Operating Characteristic Curve (AUC). Models with a TSS value of 0.7 or higher, indicative of robust performance, were selected for further analysis. To enhance the reliability of the ensemble model, individual models were weighted by their TSS scores during ensemble averaging. For estimating species distribution areas, continuous ensemble predictions were converted into binary maps. This conversion utilized the threshold that maximizes the sum of sensitivity and specificity (Liu et al. 2013, 2016), thereby ensuring that the resulting maps more accurately delineate high‐suitability areas. TD was subsequently calculated through overlap analysis of these binary maps.

We modeled distributions for 33 species with limited 2–5 records using an ensemble approach of KDE/KDE, ruLSIF and maxnet for current and future conditions (Maitner et al. 2026). This strategy mitigates model uncertainty associated with small sample sizes. A binarization threshold for outputs was derived as the 5% quantile (average ROR) of predictions at presence points. To ensure quality, models with AUC < 0.7 were discarded. Binary predictions (presence/absence) from the selected models were summed, and ensemble presence was determined by vote‐counting. The resulting prediction represents the union of all individual model outputs (any area predicted by at least one model). Analyses utilized the R package *S4DM* v0.0.1 (Maitner et al. 2025).

Potential ranges for species with only 1 record (19 species) were estimated using 5‐km buffers around occurrence points and assumed static under future scenarios.

### 2.5 Functional Diversity

We constructed a comprehensive species‐level trait database for Caudata in China, primarily based on data from Zhang et al. (2023). To fill data gaps and enhance accuracy, supporting information was integrated from Song et al. (2022) and Huang et al. (2023), as well as additional published literature (Table S3). Data were validated and refined, with corrections such as adjusting the reproductive mode of *Andrias davidianus* to external fertilization (Fan 2022), and revising the primary habitat type of *Yaotriton broadoridgus* to terrestrial (Fei 2020; Song et al. 2022). We selected 21 important traits involved in growth, reproduction and habitat selection processes (Zhang et al. 2023). To impute missing trait values, we used the *TDIP* package (Gendre et al. 2024), which integrates phylogenetic comparative models with machine learning via a hard voting (HV) ensemble.

FD was computed using the Functional Dendrogram (FDend) (Petchey and Gaston 2002). First, a functional traits matrix was converted into a Gower's distance matrix, which was then used to construct a regional functional dendrogram using hierarchical clustering of unweighted pair groups method with arithmetic mean (UPGMA). For each grid cell, the corresponding local dendrogram was extracted, and the summed branch lengths of the local dendrogram represented the FD of the grid cell. FDend is particularly suitable for capturing ecological differentiation because it can accommodate both continuous and categorical traits (Mouchet et al. 2010; Ahmed et al. 2019). The analyses were conducted at a spatial resolution of 30 arc‐seconds (~1 km × 1 km at the equator) using the *spat.alpha()* function in the R package *divraster* v1.0.4 (Mota et al. 2023).

### 2.6 Phylogenetic Diversity

To construct the phylogenetic tree for Caudata in China, we used four mitochondrial genes: *Cytb*, *Co1*, *16S‐rRNA*, and *Nd2*. Mitochondrial reference genomes for all available species were obtained from the GenBank database, and the corresponding sequences were extracted. In total, phylogenetic data for 94 species were compiled (Table S4). The species *Dermophis mexicanus* , *Hypogeophis rostratus* and *Ichthyophis kohtaoensis* were used as outgroups. The phylogenetic tree was constructed through the following steps. First, the sequences of the four genes were aligned using MEGA‐X software with MUSCLE under default parameters (Kumar et al. 2018). Next, a concatenated supermatrix was generated by combining the aligned sequences of the four genes, treating gaps as missing data, using Phylosuite v1.2.2 (Zhang et al. 2020). Bayesian inference was performed to reconstruct phylogenetic relationships using BEAST v2.6.3 (Bouckaert et al. 2014). The general time reversible (GTR) model of sequence evolution was applied across a single partition encompassing all genes, with a Yule model as the tree prior under a relaxed clock log‐normal approach. The Yule process was run for 100 million generations, and convergence was evaluated using Tracer v1.7 (Rambaut et al. 2018). A burn‐in fraction of 25% was removed before generating the final tree, which was summarized using TreeAnnotator. To estimate divergence times, three calibration points from TimeTree (Kumar et al. 2022) were applied. These calibration points corresponded to the evolutionary divergence of Caudata, the Cryptobranchoidea clade, and a group consisting of *Tylototriton*, *Echinotriton*, *Yaotriton*, and *Liangshantriton* (Figure S2).

PD was calculated as the total branch length connecting all species within a grid cell on the phylogenetic tree (Faith 1992). The PD metric also accounted for basal branch lengths shared among species, preventing zero values for single‐species communities (Rodrigues and Gaston 2002). The analyses used the *spat.alpha()* function in the R package *divraster* v1.0.4.

### 2.7 Protected Areas

Protected areas data were sourced from the China Nature Reserve Specimen Resource Sharing Platform (2024). A total of 1028 PAs vector datasets were downloaded. Additionally, all 92 PAs located in Taiwan province, which follows a unique designation system, were incorporated into the analysis. These data were downloaded from the World Database on PAs (https://www.protectedplanet.net).

### 2.8 Conservation Priority Areas of Multidimensional Biodiversity

We used Zonation 5 (Moilanen et al. 2022) to identify multidimensional conservation priority areas for Caudata in China. Zonation 5 generates complementarity‐based landscape prioritization through iterative cell‐removal optimization, preserving maximum conservation value by iteratively removing grid cells with the least value loss. For TD, we input the binary maps with presence (value = 1) as the weighting layer. For PD and FD, branch lengths from the phylogenetic tree or FDend were used as the weights. For all Zonation prioritizations, the Core‐Area Zonation algorithm (CAZ2) was used as cell remove rule, and the Human Influence Index (Sanderson et al. 2002) was integrated as a spatial constraint layer to modulate conservation value calculations. To evaluate climate change impacts, we used interaction connectivity between current and future distributions across different scenarios. In alignment with the Kunming‐Montreal Global Biodiversity Framework (CBD 2022), priority conservation areas were identified as the top 30% of grids with the highest conservation priority scores under each scenario.

### 2.9 Conservation Gap and Climatic Refugia

We evaluated congruence among diversity dimensions using two approaches: (i) Kendall rank correlation to evaluate how conservation priority values correlate across diversity dimensions, and (ii) the proportion of each diversity dimension represented within spatial priorities and their overlaps (Brum et al. 2017; Xu et al. 2019). Areas of commonality were interpreted as having the highest overall conservation priority, representing optimal priority areas that warrant focused conservation efforts. Overlaying these areas with PAs allowed us to identify key conservation gaps.

We further contrasted these conservation priority areas under different climate scenarios. To address uncertainties in predicting species distribution shifts due to climate change, we identified areas of consistently prioritized across all three climate scenarios as more robust refugia under climatic uncertainty. Such conservation gaps represent critical zones where conservation efforts should be prioritized to mitigate the impacts of climate change and protect biodiversity under future climate conditions (Wang et al. 2023). While we acknowledge potential biases inherent in the modeling process (Warren et al. 2021), our focus on areas of overlap across scenarios provides a robust foundation for identifying and protecting resilient conservation hotspots.

## 3 Results

### 3.1 Distribution Patterns of Multidimensional Diversity

**FIGURE 1:**
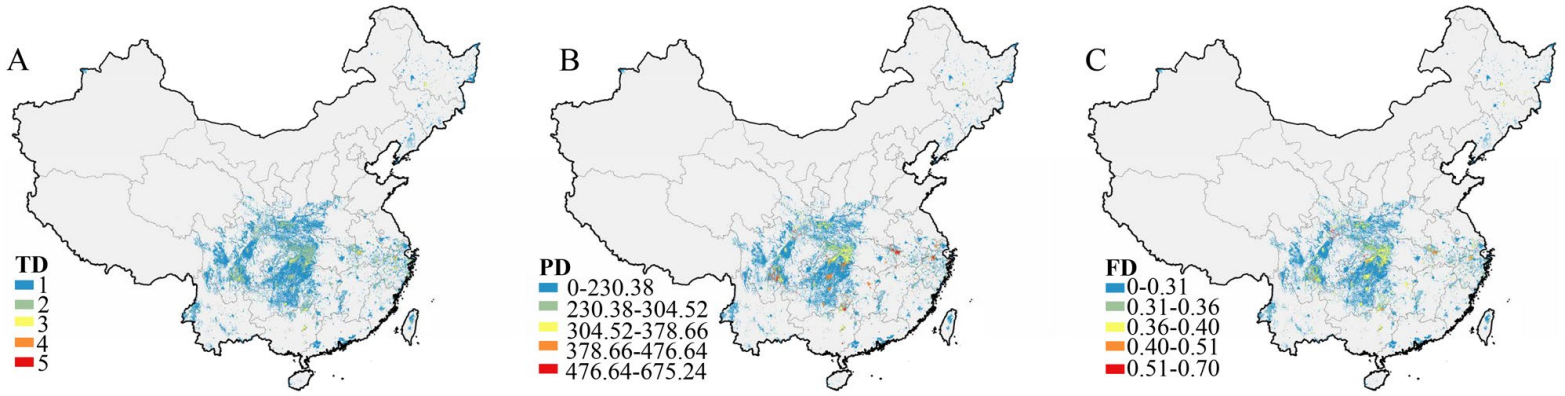
Distribution patterns of Caudata in China under the current scenario. (A) Taxonomic diversity (TD); (B) Phylogenetic diversity (PD); (C) Functional diversity (FD). Map approved under GS (2023) 2767; projection in Albers_WGS84.

Our SDMs demonstrated robust predictive performance for 101 Caudata species, with evaluation metrics of AUC = 0.92 ± 0.08 and TSS = 0.85 ± 0.11 (Table S5). The projected distributions revealed three disjunct biogeographic clusters: northeastern, northwestern, and southern China (Figure 1). PD and FD exhibited strong spatial congruence, whereas both differed considerably from TD. Kendall's concordance coefficients were τ_div = 0.99 (PD vs. FD), 0.60 (PD vs. TD), and 0.60 (FD vs. TD), indicating strong evolutionary and functional coupling among taxa. Overall, Caudata in China showed relatively low diversity, with hotspots distributed in specific mountainous and karst regions, including the Dabie Mountains in western Anhui, northern Zhejiang, and the karst landscapes of southern Sichuan and Guizhou (Figure 1). However, current PAs provided insufficient coverage of these critical zones. Among the top 1% hotspots for TD, only 9.81% were within PAs, compared to 12.57% for PD and 8.18% for FD (Table 1). Under future climate scenarios, although some species may experience range expansions, some species (27–34) were projected to face substantial habitat contraction or complete habitat loss (Table S5). Conservation vulnerability was projected to intensify in biodiversity hotspots, particularly under SSP585, where PA coverage for top 1% TD hotspots plummeted to 3.84% by 2100 (Table 1).

**TABLE 1:** Conservation effectiveness of protected areas (PAs) associated with high diversity area. Taxonomic diversity (TD); Phylogenetic diversity (PD); Functional diversity (FD).

Diversity index	Priority threshold	Current (no. of cells)	Covered by PAs	Covered by PAs under future scenarios in 2100		
				SSP126	SSP370	SSP585
TD	Top 1% (SR ≥ 3)	25,079	9.81%	5.39%	5.92%	3.84%
	Top 5% (SR ≥ 2)	160,158	9.37%	7.63%	6.85%	6.58%
PD	Top 1%	8038	12.57%	6.12%	5.37%	2.54%
	Top 5%	47,157	7.35%	4.74%	4.72%	4.17%
FD	Top 1%	11,844	8.18%	4.45%	7.50%	9.33%
	Top 5%	96,178	9.15%	6.42%	5.44%	3.28%

### 3.2 Distribution Patterns of Priority Areas Under the Current Scenario

**FIGURE 2:**
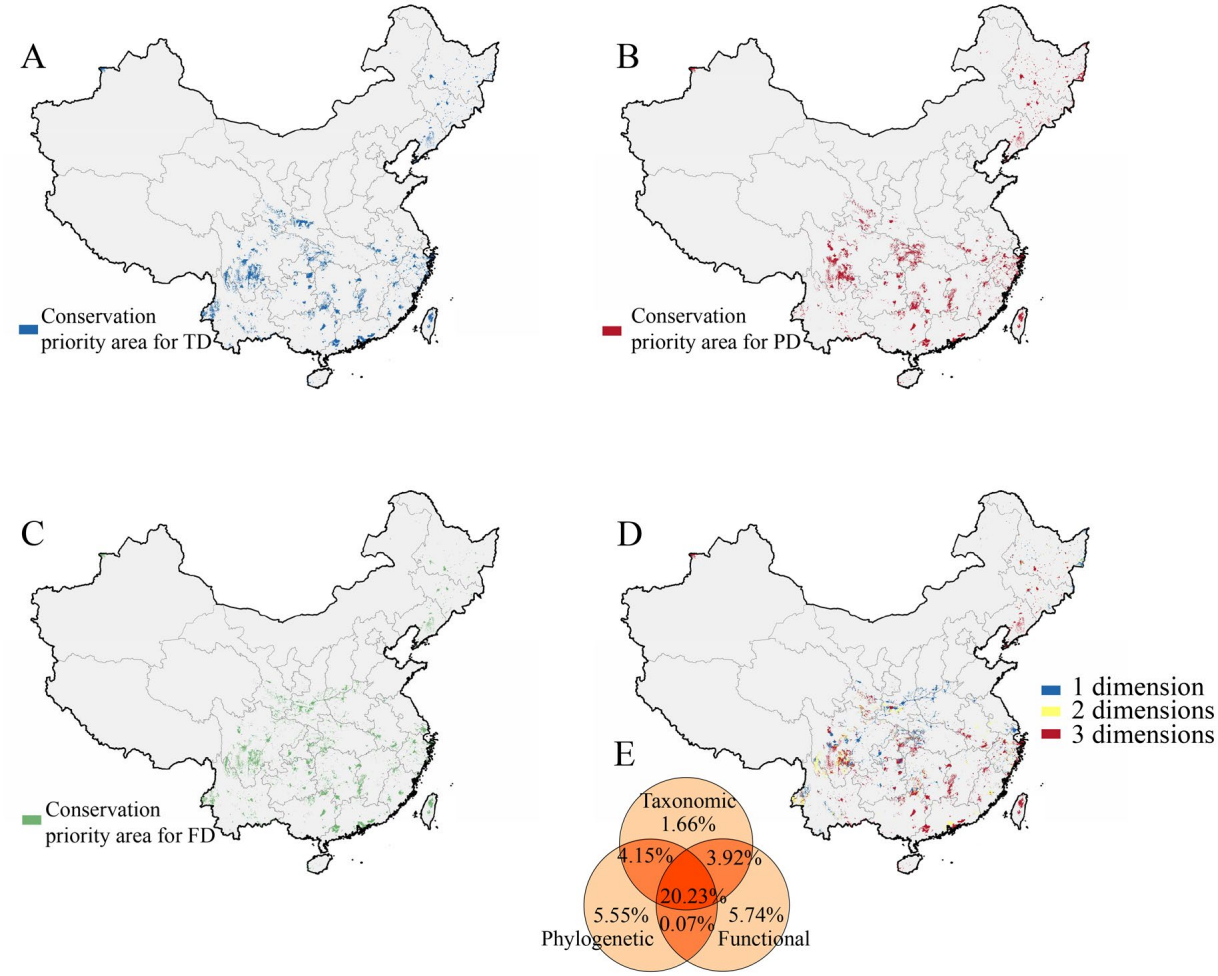
The top 30% conservation priority areas identified by zonation currently based on TD (A), PD (B), FD (C) and the overlap across the three dimensions (D), with their percentage in relation to the total distribution area of Caudata in China (E).

Our prioritizations retained 241,115 planning units per dimension, capturing 95%, 90%, and 93% of TD, PD, and FD distributions, respectively (Figure S3). Pairwise correlations between conservation values across dimensions were all positive and statistically significant, with the strongest alignment between TD and PD (Kendall's τ_prio = 0.92), whereas FD exhibited relatively weaker associations (TD‐FD: τ_prio = 0.79; FD‐PD: τ_prio = 0.73; Table S6). Notably, 24.15% of FD‐based and 24.38% of PD‐based priority areas overlapped with TD priorities, while 20.23% of grid cells were simultaneously prioritized across all three dimensions. These multidimensional priority areas clustered in biodiversity‐critical regions including the Hengduan Mountains of southwestern China, the Zhejiang–Fujian region in eastern China, the Daba Mountains, the Nanling Range, the northern Guizhou karst landscapes, Taiwan's Central Mountain Range, and transitional zones in northwestern and northeastern China. One‐ or two‐dimension priorities predominated in western Yunnan, northeastern Sichuan, and the Qinling Mountains (Figure 2). Comparative analysis revealed limited alignment between priority areas and existing PAs. Only 8.24% of TD‐based, 8.59% of PD‐based, and 7.58% of FD‐based priority areas fell within PAs, with a mere 4.44% coverage for multidimensional priority areas (Table 2).

**TABLE 2:** Percentage of conservation priority areas within PAs.

Period	Scenario	Conservation priority for three dimensions			All three dimensions
		Taxonomic	Phylogenetic	Functional	
Current		8.24%	8.59%	7.58%	4.44%
2050s	ssp126	9.36%	9.13%	8.07%	4.62%
	ssp370	9.32%	8.97%	8.21%	
	ssp585	9.33%	9.06%	8.18%	
2100s	ssp126	9.10%	8.72%	7.93%	4.67%
	ssp370	9.30%	9.02%	8.19%	
	ssp585	9.19%	9.36%	7.97%	

### 3.3 Distribution Patterns of Priority Areas Under the Future Scenarios

**FIGURE 3:**
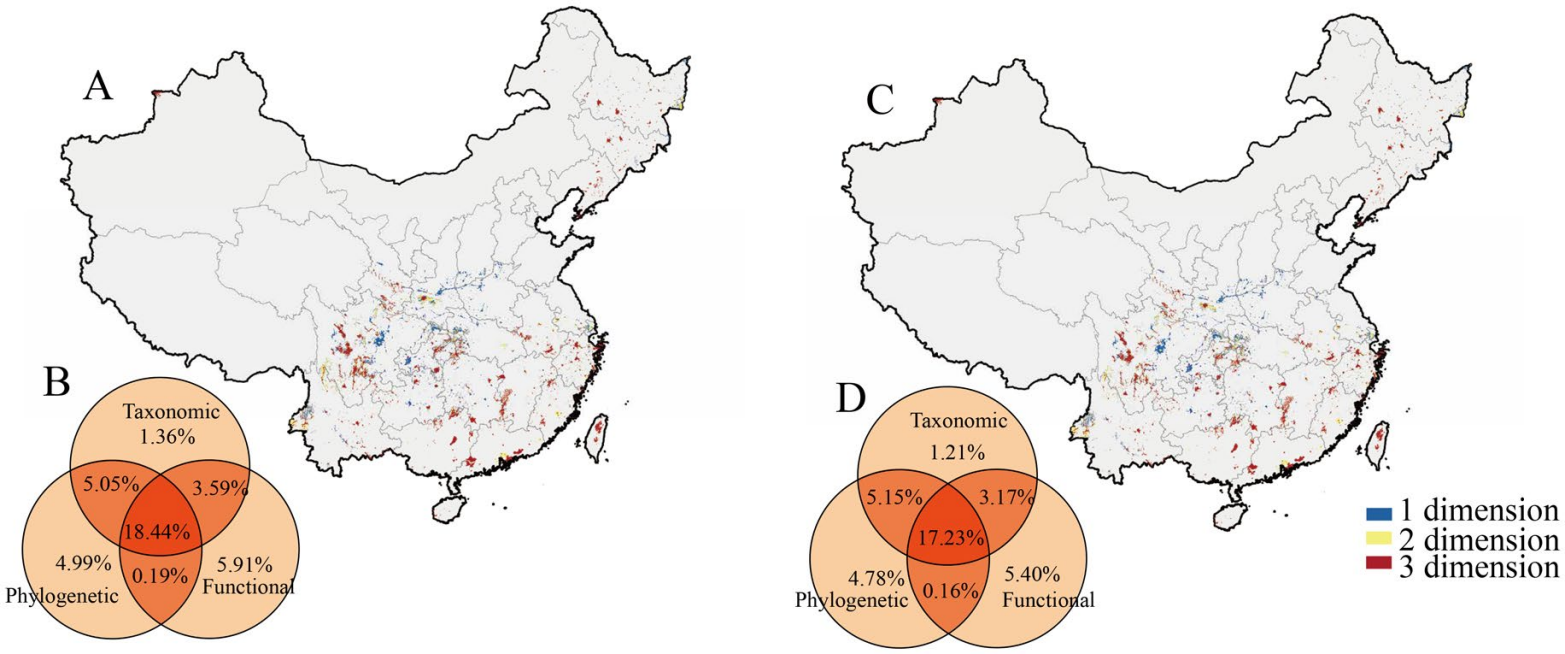
Spatial overlap (A, C) and proportional coverage (B, D) of the top 30% conservation priority areas for Caudata in China across three dimensions for the 2050s (A, B), and the 2100s (C, D).

Under future climate scenarios, conservation priority areas exhibited partial spatial shifts while maintaining high overlap (76.79%–84.99%) with current priorities (Table S7). Differences among SSP scenarios were relatively minor (Figures S4 and S5). In the 2050s, projections revealed congruence among dimensions: 22.03% of FD‐based and 23.49% of PD‐based priorities overlapped with TD‐based ones, whereas only 18.44% of grid cells were prioritized across all three dimensions (Figure 3A,B). New multidimensional priority areas were predominantly expanded into the Jinshajiang River basin in the Hengduan Mountains in southwestern Sichuan, eastern Yunnan, and the Qinling region. The one‐ and two‐dimensional priority areas also exhibited some shifts, with reductions observed in southeastern China and increases in parts of northeastern Sichuan and Chongqing. By the 2100s, three‐dimensional overlaps remained similar patterns to those of the 2050s, although the overlap of areas prioritized across all three dimensions further declined to 17.23% (Figure 3C,D). In southwestern China, the extent of one‐ and two‐dimensional priority areas diminished. Regarding overlap with existing PAs, the proportion of priority areas within PAs marginally increased under future climates. However, overall conservation effectiveness remained limited, with the proportion of priority areas within PAs still less than 10%. The protection across all three dimensions improved slightly to 4.62% in the 2050s and 4.67% in the 2100s, yet remained below 5% (Table 2).

### 3.4 Distribution Patterns of Conservation Gaps

**FIGURE 4:**
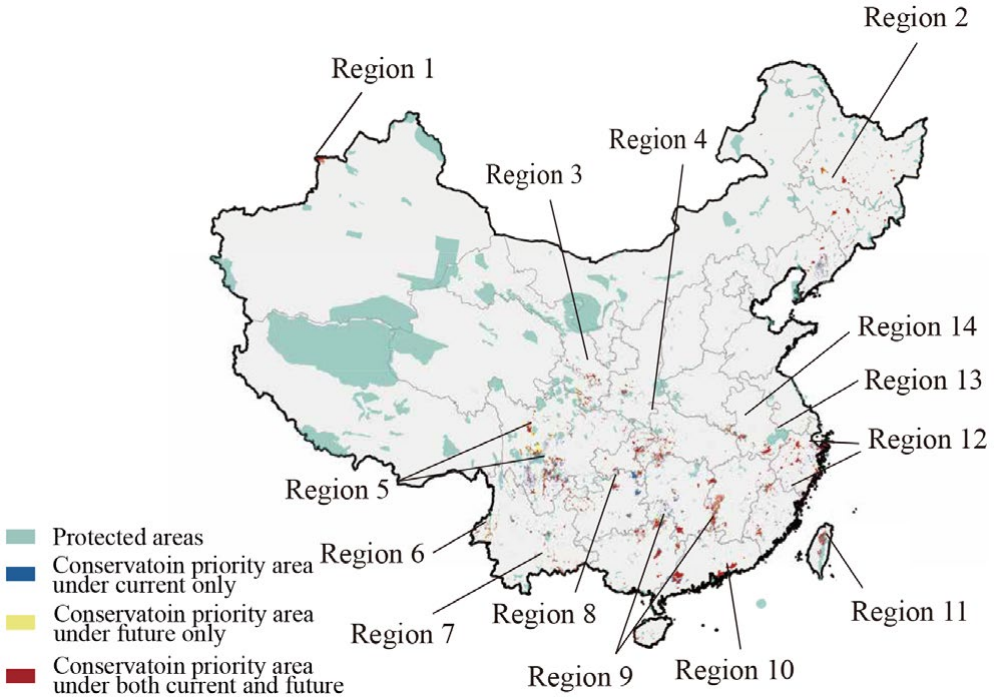
Key refugia for Caudata in China identified from the overlap of current and future conservation priority areas across climate scenarios in the 2100s, shown together with the coverage of PAs. Numbered regions indicate the major refugia identified as conservation gaps.

By overlaying current and future optimal priority areas of Caudata in China across all three climate scenarios, we found that the spatial distribution of refugia was highly consistent between the 2050s and 2100s. Among these refugia, only 9.06% and 9.04% overlapped with existing PAs in the 2050s and 2100s, respectively. Comparative analysis further identified 14 critical refugia representing major conservation gaps unprotected by the current PA network. These refugia were primarily located in the following regions: (1) Mountain steppes along the China‐Kazakhstan border in northwestern Xinjiang; (2) Montane forests of eastern Northeast China; (3) Minshan Mountains in southern Gansu; (4) Daba Mountains' transitional ecosystems; (5) Core biodiversity hotspot in the Hengduan Mountains; (6) Gaoligong Mountains in western Yunnan; (7) Ailao Mountains and Red River basin along the Yunnan‐Vietnam border; (8) Karst landscapes of northern Guizhou; (9) Nanling Mountains; (10) Pearl River basin in southern Guangdong; (11) Northern Central Mountain Range of Taiwan; (12) Zhejiang–Fujian hills in eastern China; (13) Huangshan Mountains; (14) Dabie Mountains (Figure 4 and Figure S6).

## 4 Discussion

### 4.1 Multidimensional Patterns of Caudata Diversity

This study reveals three markedly disjunct distribution patterns of Caudata across northeastern, northwestern, and southern China (Figure 1), reflecting both tectonic dynamics and climatic history. The uplift of the Qinghai‐Tibet Plateau (Favre et al. 2015) and the Asian desertification reshaped regional hydrology and topography (Zhang et al. 2006, 2008; Yao et al. 2018), promoting long‐term isolation of salamander lineages. The limited dispersal capacity and aquatic dependency of Caudata likely amplified this geographic isolation, as exemplified by localized divergence within *Batrachuperus* along the Plateau's eastern margin (Zhang et al. 2021). These patterns highlight the interplay between geological fragmentation and climatic stability, where southern refugia maintained ancestral diversity during Pleistocene oscillations, while northern disjunctions represent relict populations constrained by aridification and low connectivity.

The strong congruence between PD and FD further indicates that functional differentiation has evolved within a conserved phylogenetic framework. This pattern implies that the loss of species from evolutionarily distinct lineages may lead to a disproportionate reduction in both evolutionary history and ecological function. Such losses are particularly consequential for amphibians, whose functional roles are often closely linked to specific life‐history traits, habitat use, and trophic interactions (Hocking and Babbitt 2014). For taxa characterized by limited dispersal ability and strong microhabitat specialization, reliance on TD alone may underestimate conservation priorities by overlooking regions of high evolutionary or functional uniqueness (Faith 1992; Mazel et al. 2018). For example, the Xinjiang region, identified here as a priority area due to its high PD associated with the monotypic genus *Ranodon*, would receive limited attention under species richness–based approaches. By integrating TD, PD, and FD, our framework captures complementary facets of biodiversity and ensures that conservation strategies account not only for species richness, but also for the preservation of evolutionary lineages and ecological functions.

### 4.2 Mountains as Key Refugia for Caudata

While global PAs have demonstrated partial effectiveness in protecting amphibian biodiversity (Mi, Ma, et al. 2023), our analysis reveals significant gaps in protecting the multidimensional diversity of Caudata. Only 9.81% of top TD hotspots are currently covered, and this proportion is projected to decline under future scenarios (Table 1). By integrating multidimensional diversity with different climate scenarios, we identified 14 climatically stable refugia and conservation gaps (Figure 4). These refugia fall into three strategic configurations: (1) Global biodiversity hotspots: the Hengduan Mountains (Region 5) and southwestern Yunnan, (Regions 6 and 7) (Myers et al. 2000); (2) Hidden hotspots of amphibian diversity in China: Nanling Mountains (Region 9), Wuling‐Tianmu ranges (Region 13) (Xu et al. 2024); (3) Evolutionarily irreplaceable sites for Caudata in China: Xinjiang (Region 1), Northeast China (Region 2), Minshan Mountains (Region 3), and Dabie Mountains (Region 14) (Yao et al. 2018). Together, these areas show that the most important refugia for Caudata are not restricted to the richest regions alone, but also include climatically persistent and evolutionarily distinctive areas that would be undervalued by richness‐based approaches. The Xinjiang region, in particular, has often been overlooked in previous studies on amphibians (Zhang et al. 2023; Xu et al. 2024). However, *Ranodon sibiricus* , which is distributed in this area, belongs to an ancient monotypic genus. Within China, only six populations of this species have been identified, and its genetic diversity is extremely low, highlighting the urgent need for targeted conservation efforts (Chen et al. 2012).

These findings underscore the indispensable role of mountain ecosystems in protecting Caudata in China under climate change. The identification of 14 climatically stable refugia suggests that mountain systems function not only as centers of current diversity, but also as spatial buffers against future climatic change. With their complex topography and microclimatic diversity, mountainous regions create habitat heterogeneity that can buffer biodiversity from climate impacts (Rahbek et al. 2019). This buffering capacity is closely associated with thermal differentiation across elevational gradients, which helps preserve suitable microhabitats and may facilitate short‐distance climate tracking for species with limited dispersal. Globally, mountains support 85% of terrestrial vertebrate species, serving as critical biodiversity reservoirs (Rahbek et al. 2019). For Caudata, mountain systems provide essential ecological functions, hydrological continuity, and connectivity corridors that maintain genetic exchange between isolated populations (Luo et al. 2021; Yang et al. 2022). However, these biologically rich areas face escalating threats from habitat fragmentation and climate‐driven stressors (Albrich et al. 2020; Chan et al. 2024). As climate change intensifies, prioritizing the conservation of elevational gradients and headwater networks within mountain refugia will be crucial to enhance species resilience and to mitigate extinction risks.

### 4.3 Management Implications

The synergistic pressures of climate change and habitat fragmentation demand a shift in amphibian conservation. Given that Caudata species generally exhibit limited dispersal abilities, strong dependence on microhabitats, and high sensitivity to localized environmental changes (Nowakowski et al. 2017), conventional static PA frameworks may be insufficient in the face of escalating climate pressures and anthropogenic perturbations. Our results suggest that the identified refugia should be incorporated into conservation planning as priority areas for climate‐adaptive protection. In particular, different strategic configurations of refugia may require different conservation approaches. Global biodiversity hotspots such as the Hengduan Mountains and southwestern Yunnan should be prioritized for PA expansion along complete elevational gradients, so that species can track suitable climatic conditions over relatively short distances as temperatures shift (Rahbek et al. 2019). Hidden amphibian hotspots and evolutionarily irreplaceable regions may instead require more targeted site‐based protection for localized populations and habitats, especially where species are highly range‐restricted or represent deep evolutionary lineages. Across all configurations, enhancing ecological connectivity among mountain refugia should be a conservation priority, particularly through the protection of headwater habitats, riparian systems, and associated forested corridors that can maintain hydrological continuity and facilitate dispersal and genetic exchange (Luo et al. 2021; Yang et al. 2022). Because many of the identified refugia fall outside existing PAs, future regional planning should also integrate dynamic climate projections to identify climatically persistent areas before they are lost to land‐use change (Wang et al. 2023). At the same time, other effective area‐based conservation measures (OECMs), such as watershed protection forests and community‐managed mountain landscapes, may provide an important complement to PAs in improving protection coverage and maintaining local microhabitats (Chen et al. 2024; Cook 2024).

Several limitations should nevertheless be acknowledged. First, SDMs are inherently probabilistic, and projections under future climate change depend on assumptions related to climate scenarios, predictor choice, model structure, and the transfer of models to novel environmental conditions (Warren et al. 2021). Second, species with very limited occurrence data required alternative modeling approaches, and single‐record species were treated conservatively (Maitner et al. 2025, 2026). While this allowed rare species to be retained in the analysis, it may have simplified their future range dynamics and increased uncertainty in projected suitable areas. Third, the refugia identified in this study should be interpreted as robust spatial priorities derived from overlap among current and future conservation priority areas across climate scenarios, rather than as exact forecasts of future species persistence. Future work that incorporates finer‐scale microclimatic information, dispersal constraints, and land‐use change dynamics would further improve climate‐adaptive conservation planning for Caudata (Warren et al. 2021; Wang et al. 2023). Nevertheless, the refugia identified here remain valuable priorities for climate‐adaptive conservation planning for Caudata in China.

## 5 Conclusion

This study emphasizes the inadequacy of China's current PA network in protecting the multidimensional biodiversity of Caudata under climate change. By integrating taxonomic, phylogenetic, and functional data, we reveal significant spatial mismatches between conservation priorities and existing PAs, as well as low overlap among key biodiversity dimensions. Future climate scenarios indicate worsening protection, particularly under high‐emission pathways. The identification of 14 refugia offers valuable guidance for conservation expansion, especially in mountainous regions that act as climate buffers. To enhance conservation effectiveness, we recommend incorporating these refugia into regional biodiversity strategies, advancing dynamic spatial planning, and leveraging both formal and complementary area‐based conservation measures. Our multidimensional framework provides a practical basis for prioritizing climate‐vulnerable and data‐poor taxa in future conservation planning.

## Author Contributions

**Lu‐Lu Sui:** data curation (equal), formal analysis (equal), methodology (equal), writing – review and editing (equal). **Bin Wang:** data curation (equal), writing – original draft (equal). **Feng Xie:** data curation (equal), writing – original draft (equal). **Wei Zhu:** data curation (equal), writing – original draft (equal). **Cheng Shen:** data curation (equal), writing – original draft (equal). **Jian‐Ping Jiang:** data curation (equal), writing – original draft (equal).

## Funding

This work was supported by the National Key Programme of Research and Development, Ministry of Science and Technology (2022YFF1301401), Report on the Survival Status of Endangered Species in China: Assessment Report on the Endangered Status of Tailed Amphibians in China, and China Biodiversity Observation Network (Sino BON—Amphibian & Reptile).

## Conflicts of Interest

The authors declare no conflicts of interest.

## Supporting information


**Table S1:** List of Caudata amphibian in China.
**Table S2:** Sources of species occurrences.
**Table S3:** Compiled functional traits.
**Table S4:** Sequence used in phylogenetic analysis.
**Table S5:** Model accuracy, threshold and suitable areas.
**Table S6:** Kendall rank correlation coefficients (τ) between the conservation priority values given by the spatial prioritization analysis for each pair of dimensions.
**Table S7:** Percentage of overlap between conservation priority areas under different climate scenarios and current scenario.

**Figure S1:** Pearson's correlation coefficient between each pair of environmental variables. Alt, altitude; GSL, growing season length; HF, human footprint; NPP, net primary productivity; PD, human population density; PFT, plant functional types; UVBH, mean UV‐B of highest month; UVBL, mean UV‐B of lowest month.
**Figure S2:** Phylogenetic tree of 94 Caudata species in China constructed from *Cytb, Co1, 16S‐rRNA*, and *Nd2* data.
**Figure S3:** Performance curves of Zonation prioritization showing the proportion of retained planning units and biodiversity representation across TD, PD, and FD.
**Figure S4:** Top 30% conservation priority areas for Caudata in China in the 2050s identified by Zonation under future climate scenarios based on TD (A), PD (B), and FD (C).
**Figure S5:** Top 30% conservation priority areas for Caudata in China in the 2100s identified by Zonation under future climate scenarios based on TD (A), PD (B), and FD (C).
**Figure S6:** Key refugia for Caudata in China identified from the overlap of current and future conservation priority areas across climate scenarios in the 2050s, shown together with the coverage of PAs.

## Data Availability

Data used for this analysis are publicly available. Environmental datasets were obtained from multiple open‐access sources, which are listed and described in the manuscript. R packages employed in the analyses have been cited accordingly. All code is publicly available via Figshare (https://doi.org/10.6084/m9.figshare.31869385). Corrected species occurrence data supporting the findings of this study were available from the corresponding author upon reasonable request.
