# Supplementary material for: Multidimensional Diversity Identifies Mountains as Key Refugia for Caudata in China
Source: Ecol Evol. 2026 May 13;16(5):e73497. doi: 10.1002/ece3.73497 (PMC13172278; doi:10.1002/ece3.73497)
Supplement: Supplementary file 2 — Figure S1: Pearson's correlation coefficient between each pair of environmental variables. Alt, altitude; GSL, growing season length; HF, human footprint; NPP, net primary productivity; PD, human population density; PFT, plant functional types; UVBH, mean UV‐B of highest month; UVBL, mean UV‐B of lowest month. Figure S2: Phylogenetic tree of 94 Caudata species in China constructed from Cytb, Co1, 16S‐rRNA, and Nd2 data. Figure S3: Performance curves of Zonation prioritization showing the proportion of retained planning units and biodiversity representation across TD, PD, and FD. Figure S4: Top 30% conservation priority areas for Caudata in China in the 2050s identified by Zonation under future climate scenarios based on TD (A), PD (B), and FD (C). Figure S5: Top 30% conservation priority areas for Caudata in China in the 2100s identified by Zonation under future climate scenarios based on TD (A), PD (B), and FD (C). Figure S6: Key refugia for Caudata in China identified from the overlap of current and future conservation priority areas across climate scenarios in the 2050s, shown together with the coverage of PAs. [file ECE3-16-e73497-s001.docx]

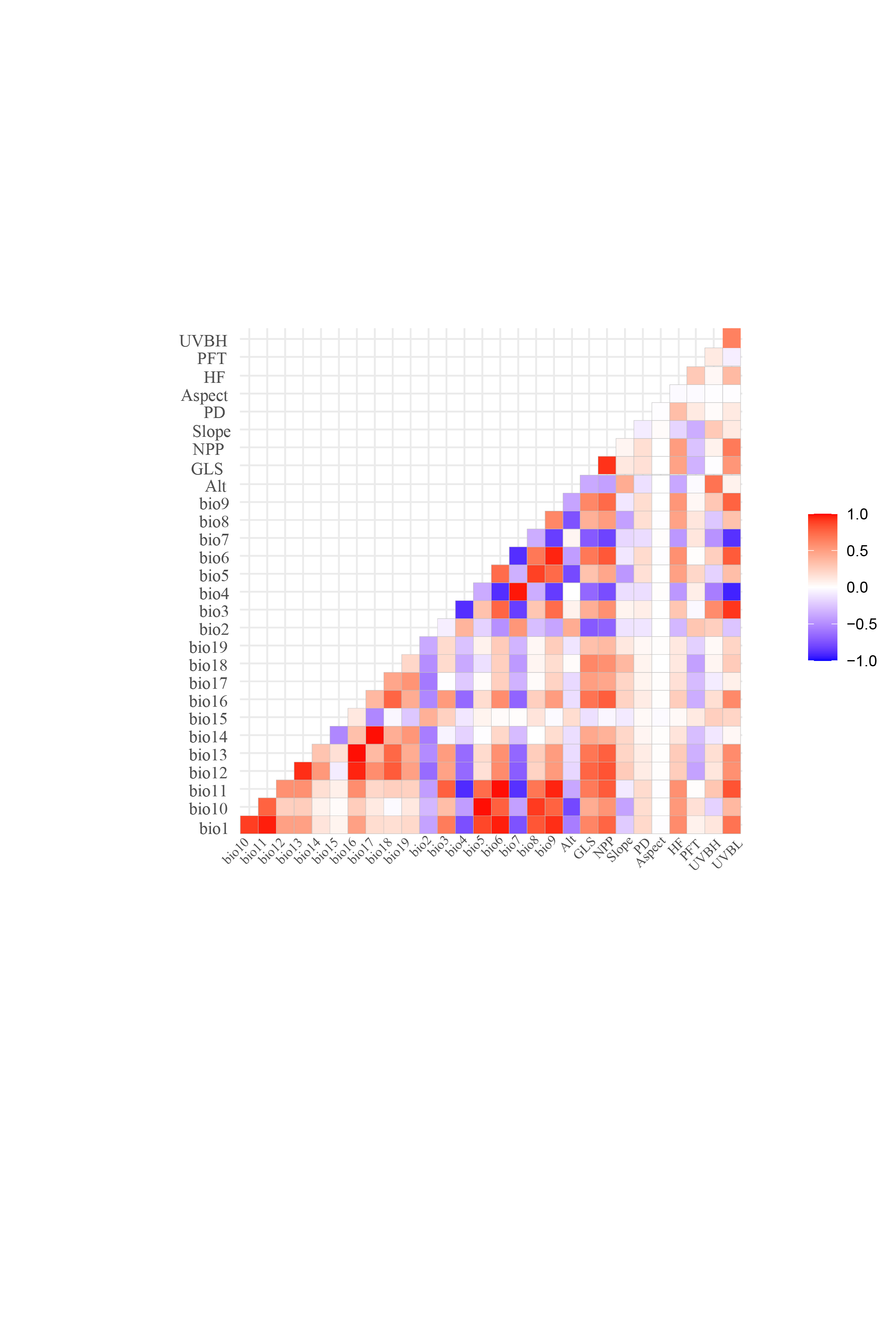


Figure S1 Pearson’s correlation coefficient between each pair of environmental variables. UVBH, mean UV-B of highest month; UVBL, mean UV-B of lowest month; PFT, plant functional types; HF, human footprint; PD, human population density; Alt, altitude; GSL, growing season length; NPP, net primary productivity.


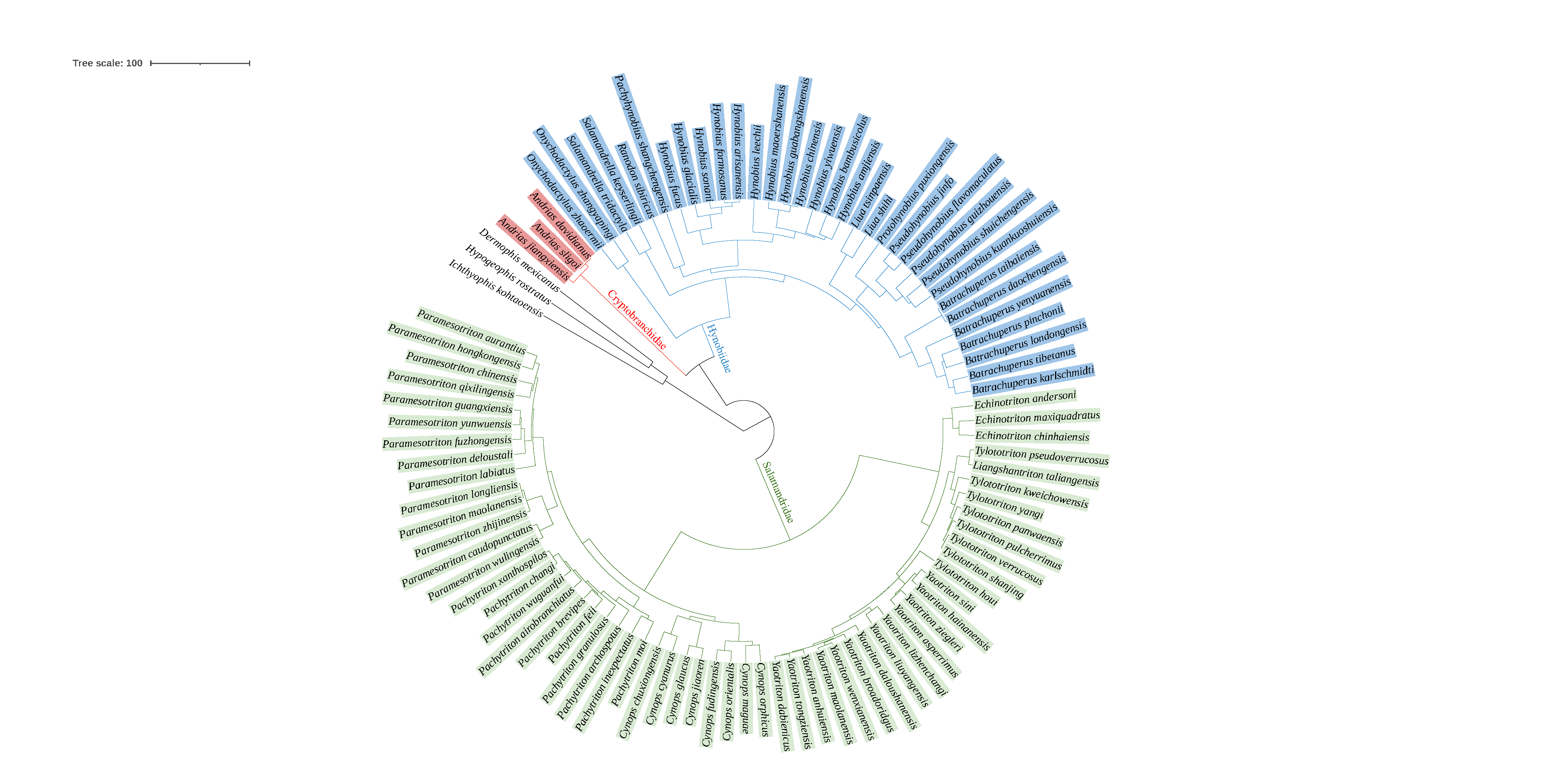


Figure S2 Phylogenetic tree of 94 Caudata species in China constructed from *Cytb, Co1, 16S-rRNA,* and *Nd2* data.


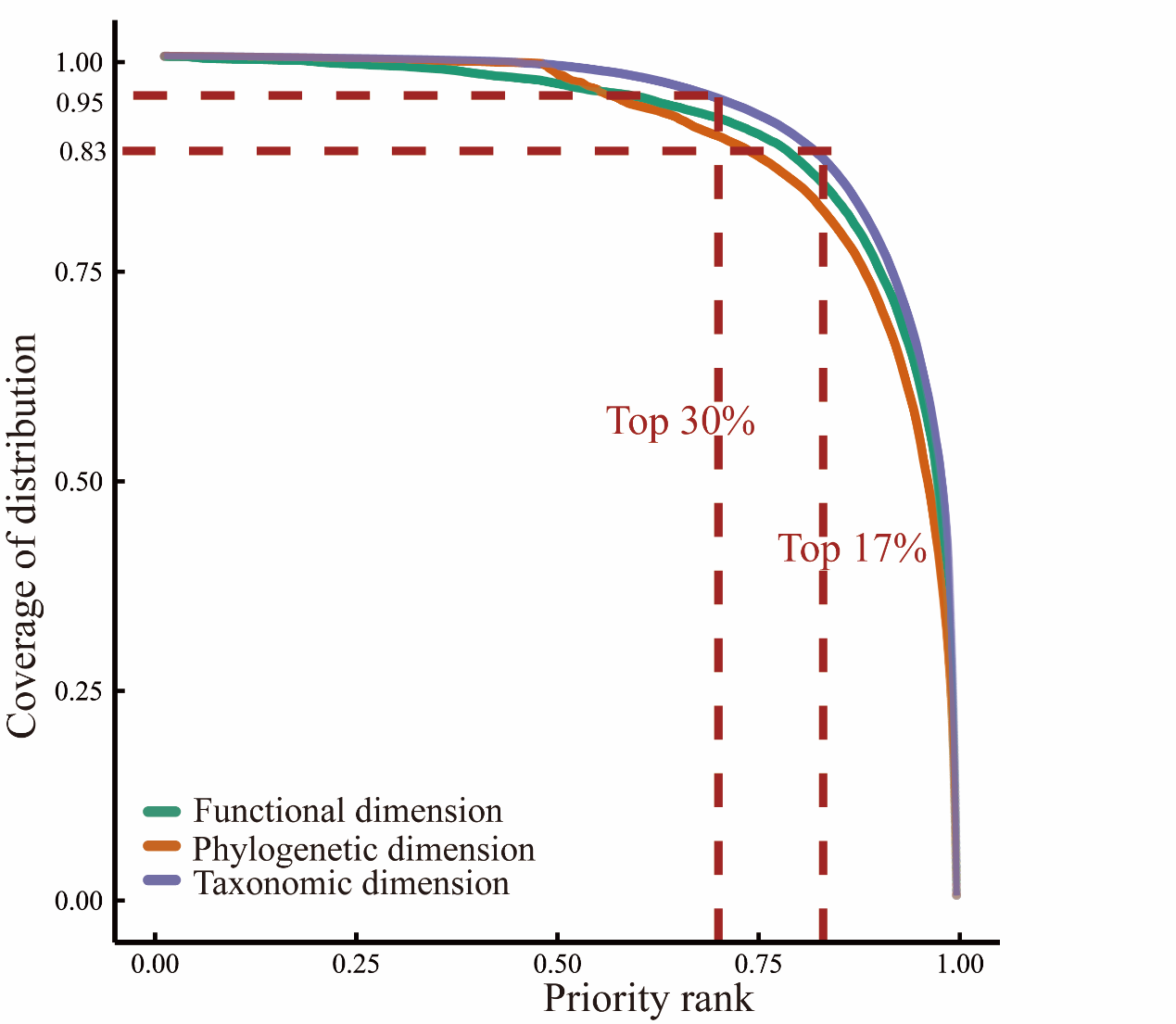


Figure S3 Performance curves of Zonation prioritization showing the proportion of retained planning units and biodiversity representation across TD, PD, and FD.


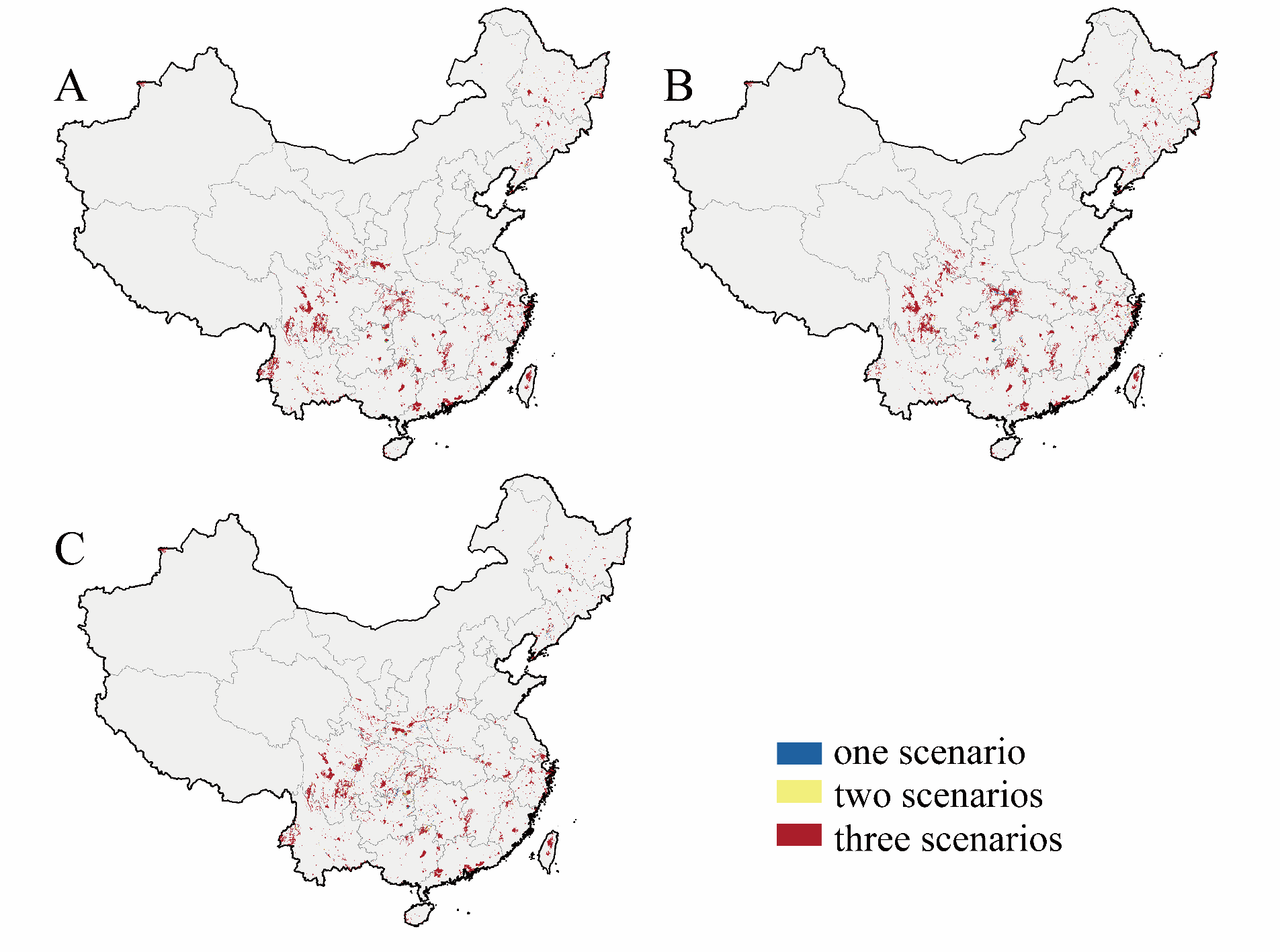


Figure S4 Top 30% conservation priority areas for Caudata in China in the 2050s identified by Zonation under future climate scenarios based on TD (A), PD (B), and FD (C).


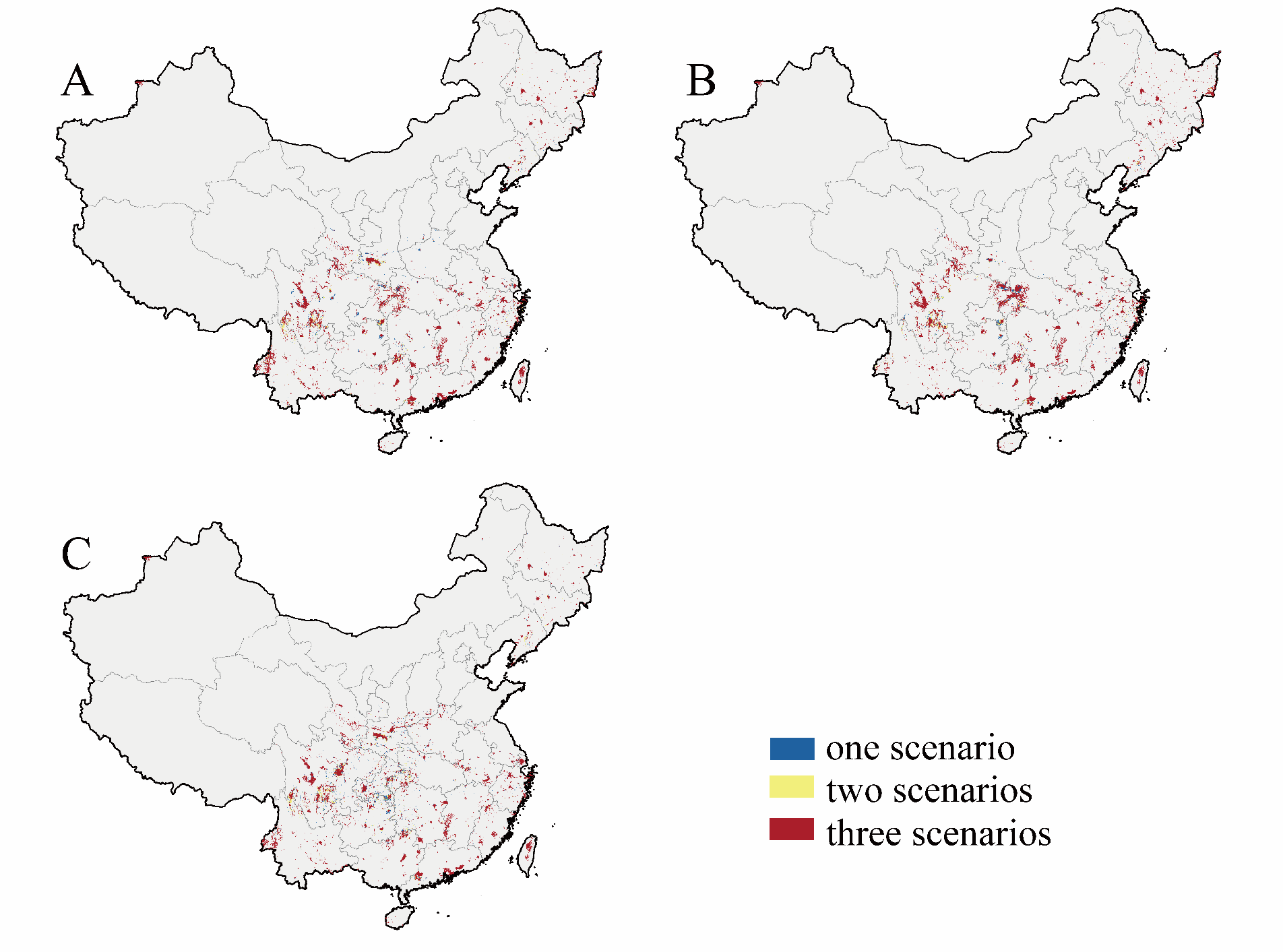


Figure S5 Top 30% conservation priority areas for Caudata in China in the 2100s identified by Zonation under future climate scenarios based on TD (A), PD (B), and FD (C).


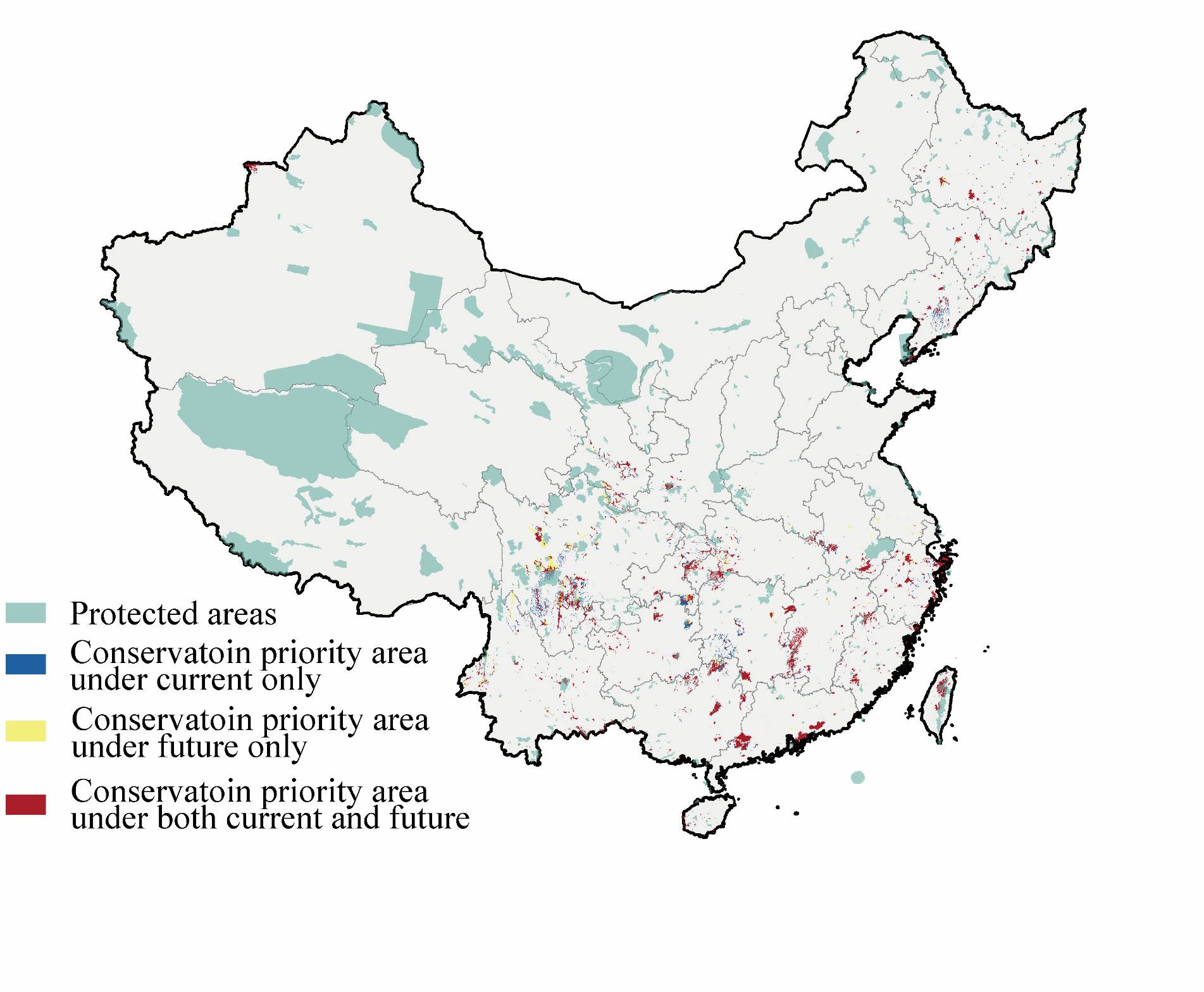


Figure S6 Key refugia for Caudata in China identified from the overlap of current and future conservation priority areas across climate scenarios in the 2050s, shown together with the coverage of PAs.
